# Key Factors in Effective Patient-Tailored Dosing of Fluoroquinolones in Urological Infections: Interindividual Pharmacokinetic and Pharmacodynamic Variability

**DOI:** 10.3390/antibiotics11050641

**Published:** 2022-05-11

**Authors:** Oskar Estradé, Valvanera Vozmediano, Nerea Carral, Arantxa Isla, Margarita González, Rachel Poole, Elena Suarez

**Affiliations:** 1 Department of Urology, Cruces University Hospital, 48903 Barakaldo, Spain; oskarestrade@gmail.com; 2 Center for Pharmacometrics and Systems Pharmacology, Department of Pharmaceutics, University of Florida, Gainesville, FL 32612, USA; valva@cop.ufl.edu (V.V.); margonzalez@ufl.edu (M.G.); poole0722@ufl.edu (R.P.); 3 Department of Pharmacology, Faculty of Medicine and Nursey, University of Basque Country UPV/EHU, 48940 Leioa, Spain; nereakarral@gmail.com; 4 Biocruces Health Research Institute, 48903 Barakaldo, Spain; 5 Pharmacokinetic, Nanotechnology and Gene Therapy Group (PharmaNanoGene), Faculty of Pharmacy, Centro de Investigación Lascaray Ikergunea, University of the Basque Country UPV/EHU, 01006 Vitoria-Gasteiz, Spain; arantxa.isla@ehu.eus; 6 Instituto de Investigación Sanitaria Bioaraba, Microbiology, Infectious Disease, Antimicrobial Agents, and Gene Therapy, 01006 Vitoria-Gasteiz, Spain

**Keywords:** fluoroquinolone, interindividual variability, pharmacokinetic, pharmacodynamic

## Abstract

Fluoroquinolones (FQs) are a critical group of antimicrobials prescribed in urological infections as they have a broad antimicrobial spectrum of activity and a favorable tissue penetration at the site of infection. However, their clinical practice is not problem-free of treatment failure, risk of emergence of resistance, and rare but important adverse effects. Due to their critical role in clinical improvement, understanding the dose-response relation is necessary to optimize the effectiveness of FQs therapy, as it is essential to select the right antibiotic at the right dose for the right duration in urological infections. The aim of this study was to review the published literature about inter-individual variability in pharmacological processes that can be responsible for the clinical response after empiric dose for the most commonly prescribed urological FQs: ciprofloxacin, levofloxacin, and moxifloxacin. Interindividual pharmacokinetic (PK) variability, particularly in elimination, may contribute to treatment failure. Clearance related to creatinine clearance should be specifically considered for ciprofloxacin and levofloxacin. Likewise, today, undesired interregional variability in FQs antimicrobial activity against certain microorganisms exists. FQs pharmacology, patient-specific characteristics, and the identity of the local infecting organism are key factors in determining clinical outcomes in FQs use.

## 1. Introduction

Fluoroquinolones (FQs) are considered a critically important antimicrobial class to human medicine [1]. They have a broad spectrum of activity against numerous Gram (+) and Gram (-) bacteria and exhibit favorable pharmacokinetic properties that facilitate adequate drug disposition at the site of infection. Due to these properties, FQs have been used to treat different types of systemic infections both in the outpatient and inpatient setting [2,3], and also a variety of urological infections, such as pyelonephritis, urethritis, and bacterial prostatitis [4]. Especially in the latter, their role is essential to saving lives. However, two clear problems currently exist in clinical practice in the management of urological infections with FQs: the emergence of uropathogen resistance [5,6] and the wide array of reported adverse effects [7,8]. Consequently, their use has been limited to strictly necessary situations in clinical practice [7,8,9].

The most commonly used FQs in urological practice in the United States and Europe are ciprofloxacin (CIP), levofloxacin (LEV), and moxifloxacin (MOX) [2,3,10]. The recommended doses are 250 to 500 mg orally or 400 mg intravenously for CIP, 500 to 750 mg orally or intravenously for LEV, and 400 mg orally or intravenously for MOX [2,3,4]. In general, these dosing regimens allow adequate drug distribution at the site of infection to achieve sufficient antibiotic exposure and treatment efficacy. However, the use of standard empirical doses may lead to unnecessary overexposure and a higher incidence of adverse effects in some patients and, consequently, non-compliance or discontinuation of drug treatment. In contrast, some patients could experience underexposure, risking treatment failure and the development of bacterial resistance [11,12,13].

**Figure 1:**
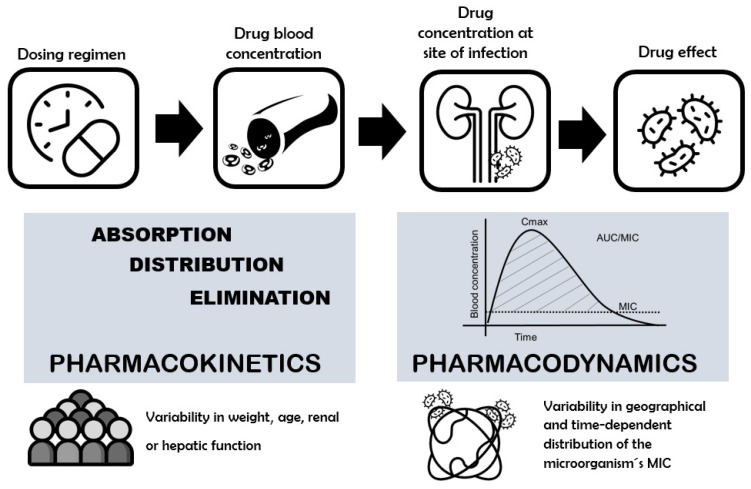
Pharmacokinetic/Pharmacodynamic factors affecting the dose-antimicrobial response relation. Cmax: peak plasma concentration, AUC: area under the plasma drug concentration-time curve from timcentration-time curve from time, MIC: minimum inhibitory concentration of an antibiotic against a bacterial pathogen.

Optimally dosing FQs is dependent on several factors, such as pathophysiological characteristics of the patient, the infecting organism, the site of infection, and the pharmacokinetic/pharmacodynamic (PK/PD) properties of the drug (Figure 1). PK properties include the factors affecting drug absorption, distribution, metabolism, and elimination, which determine its concentration in the body. Physiopathological factors associated with the patient may be responsible for the interindividual variability of the PK processes. PD describe the mechanism by which the drug exerts its antimicrobial effect [14,15]. Interregional and time-dependent differences in MIC distribution values are responsible for the PD variability of the antibiotic. Careful consideration of the factors affecting PK/PD should allow for selection of the most appropriate antibiotic when treating urological infections and establishing the dosage with a better risk/benefit ratio in terms of efficacy, safety, and development of resistances [8,13,16,17]. The interindividual variability in PK/PD processes is likely to be one of the main contributors to the variability in the antibacterial dose-exposure response relation [18,19].

The objective of the current review is to identify sources of interindividual variability in PK/PD properties of the most commonly FQs used in urological practice–CIP, LEV and MOX–which could influence the antibacterial dose-exposure response relation. Evaluation of their effect on FQs clinical outcomes would allow the development of patient-tailored dosing strategies, leading to reduced treatment failure, especially in critical infections as prostatitis.

## 2. Concerns about the Clinical Efficacy of Fluoroquinolone Dosing: The Role of PK/PD Index as a Tool

Selecting a dose to guarantee antimicrobial efficacy while minimizing the risk of resistance emergence with minimum adverse effects should be based on the FQ’s PK properties, PD properties, and the probability to attain the PK/PD index associated with clinical efficacy with the administered dose [14,16,20,21]. PK properties refer to drugs’ absorption, distribution, metabolism, and excretion, while PD properties are related to the potential activity of an antibiotic against a bacterial pathogen, measured as the minimum inhibitory concentration (MIC), and postantibiotic effect. The PK/PD target is the key value associated with antimicrobial efficacy and varies according to a chosen endpoint such as stasis, maximal kill, or resistance suppression for preclinical studies, and microbiological or clinical cure for clinical studies [15,16,22]. To attain a favorable PK/PD target for FQ, pathogens must have adequate antimicrobial exposure based on their MIC. This exposure, measured as peak plasma concentration (Cmax) and area under the plasma drug concentration-time curve from time 0–24 h (AUC_0–24h_), is dependent on the dose used and the PK properties of the drug. Only the unbound drug concentrations are microbiologically active, and therefore, the PK/PD index should be based on free drug concentrations [21].

A substantial number of studies have been performed to identify the PK/PD index relation associated with the bactericidal activity and clinical efficacy of FQs. Antimicrobial activity of FQs exhibits concentration-dependent killing along with prolonged persistent effects. Multiple clinical and preclinical data suggest that the ratio of free AUC_0–24h_ (*f*AUC_0–24h_) to MIC (*f*AUC_0–24h_/MIC) is the best PK/PD index to link antimicrobial disposition, the MIC value, and the clinical efficacy of FQs [15,23,24]. While the ratio Cmax/MIC outweighs the ratio *f*AUC_0–24h_/MIC as an indicator of resistance suppression, fewer studies exist with the aim to identify drivers for resistance suppression [11].

Based on the above concepts, Monte Carlo simulations (MCS) can be run to computationally estimate the likelihood of a given drug dose to attain a predefined value of a PK/PD target previously defined for urological FQs [19,21]. The probability of target attainment (PTA)–defined as the probability that a specific value of the PK/PD index associated with the efficacy of the antibiotic is achieved at a certain MIC [20]–can therefore be calculated for different MIC values against a variety of pathogens. Thus, PK/PD indexes can be used as a tool to select dosing regimens with PTA >90% in the studied population, increasing the probability of selecting clinically successful treatments, identifying clinical breakpoints, and preventing the emergence of resistance [14,19].

The use of these metrics is therefore essential to streamline FQs treatment and adjust dosing regimens in clinical practice. In addition, the individual status of the patient and the suspected infecting organism should be accounted for in dose decision-making. In an exposure–response model based on clinical data from patients with community-acquired pneumonia associated with S. pneumoniae, Gram (+) microorganism, and treated with a 500 mg oral dose daily of LEV, the probability of successful clinical response was 95% in patients who achieved a target of *f*AUC_0–24h_/MIC > 33.8, and was 67% in patients who did not achieve that target [23]. For infections caused by Gram (-) bacteria, the threshold of the ratio AUC_0–24h_/MIC was found to be a significant breakpoint for probabilities of both clinical and microbiologic cures, but the required value for treatment success was higher than that required for Gram (+) bacterial infections. In hospitalized patients treated with a 400 mg intravenous dose of CIP, at AUC_0–24h_/MIC > 125, the probabilities of clinical and microbiological cure were 80% and 82%, respectively, for Gram (-) bacteria [25]. Similar results were obtained for patients with nosocomial pneumonia and Gram (-) isolates treated with a 750 mg intravenous dose of LEV [24]. The United States Committee on Antimicrobial Susceptibility Testing (USCAST) report provides an extensive integrative evaluation of the in vitro susceptibility testing, PK/PD breakpoints (i.e., AUC_0–24h_/MIC ratio targets for *Enterococcus* spp., *Escherichia coli,* and other *Enterobacteriaceae* spp., *Pseudomonas aeruginosa*, and *Streptococcus aureus*), and clinical breakpoints by infection type [26]. However, similar studies in patients with urological infections are still scarce.

Published PK/PD studies have raised concerns about patient clinical outcomes with the use of FQs, particularly with regards to treatment failure after empiric dosing related to variability in PK and bacteria susceptibility, as MIC value. Rattanaumpawan et al. [27] examined the impact of PD variability associated with MIC value on FQs’ clinical outcomes in adult female patients with complicated urinary tract infections caused by *E. coli*. Treatment failure rates of 0.8% and 6.9% were observed when they compared isolates with low and high MIC values, respectively. Peloquin et al. [28] found that resistance to CIP occurred in patients after treating nosocomial lower respiratory tract infections caused by Gram (-) bacteria, such as *P. aeruginosa*. The authors explained that the Cmax/MIC ratio was far greater with isolates that were eradicated than with those that persisted, attributing variability in MIC values as the determining factor in treatment resolution. In patients with nosocomial pneumonia, measuring CIP and LEV concentrations, determining pathogen MIC, and subsequently performing dose adjustments significantly improved the probability of successful clinical outcomes and pathogen eradication [29]. For patients diagnosed with *Mycoplasma genitalium*, a pathogen transmitted through sexual contact, the increased use of 400 mg of MOX caused an emergence of cases with treatment failure. The MIC of MOX in the mutant strains increased 4-fold as compared with that of the parent strain [30].

Noreddin et al. [31] determined that age plays an important role in explaining the interindividual variability in the PK of hospitalized patients with community-acquired pneumonia treated with LEV. This factor influenced the ability to achieve the target attainment related with clinical response. Variations in clinical response to S. pneumoniae were observed when comparing elderly and younger patients. Elderly patients showed higher AUC_0–24h_ values leading to a higher AUC_0–24h_/MIC ratio and improved bacteriological outcome compared to younger adults. In another study analyzing CIP use in hospitalized patients with urinary tract, abdominal, and various other infections produced by Gram (-) microorganisms, 21–75% of the patients did not achieve the efficacy target of AUC_0–24h_/MIC ratio ≥125 with MICs of 0.25 and 0.5 mg/L, respectively. The AUC_0–24h_ achieved with standard dosing was related to the high interindividual variability of CIP clearance, associated with age and serum creatinine. This pharmacokinetic variable and the elevated MIC values observed in this study highlight the need for individualizing dosing regimens to maximize efficacy, minimize adverse effects, and prevent the appearance of resistance [32].

Ongoing efforts are still need to identify optimal FQs dosing strategies to achieve the efficacy target of AUC_0–24h_/MIC during urological clinical use due to the critical role that the management of these drugs plays in this type of infections [2,12,21]. Interindividual variability in FQs PK and urological microorganism MIC needs to be known and considered during dose adjustment in patients, as its variability can negatively influence the probability of reaching the efficacy PK/PD index and achievement of successful clinical outcomes [33,34].

## 3. Interindividual Pharmacokinetic Variability and Their Causes

Antimicrobial exposure related to drug disposition, given as AUC_0–24h_ and Cmax, is subject to interindividual variability in PK properties, such as drug absorption, distribution, and elimination. Table 1, Table 2 and Table 3 summarize the PK parameters and their important interindividual variability for CIP, LEVO, and MOX, respectively, in different patient populations extracted from literature. It is important to consider that the estimation of individual PK parameters for each patient allows the estimation of individual exposures to the drug; therefore, population PK studies are essential to achieve this objective [18,19,33,34]. In the following sections, the factors identified as potential sources of variability in the PK processes of FQs are discussed (absorption, distribution, and elimination).

### 3.1. Absorption Process: Role of Food

Absorption refers to the amount of drug reaching the bloodstream from the site of administration. FQs are well absorbed after oral administration with bioavailability (F) values of 70% for CIP [35], 99% for LEV [36], and 86% for MOX [37]. Concomitant oral administration of antacids containing multivalent cations, such as calcium, aluminum, or magnesium, calcium or iron supplements, and sucralfate, decrease FQs absorption due to the formation of insoluble quinolone-multivalent cation chelates in the gastrointestinal tract. For example, for CIP, F decreases to 15% with concomitant aluminum and magnesium antacid use within 5 to 10 min of drug administration [38]. Similar effects have been reported for milk, other dairy products, and supplements containing multivalent cations. The extent of the interaction diminishes when the interacting drug is administered at least 2 to 4 h before or 6 to 8 h after the FQs [39]. Multivalent cations present in food, supplements, or other drug products can lead to clinically relevant interactions with FQs, contributing to variability in drug absorption, reducing the overall exposure, and increasing the risk for therapeutic failure. Conversely, food not containing multivalent cations is not expected to modify FQs absorption [39,40].

According to the Food and Drug Administration’s Biopharmaceutics Classification System (BCS), CIP is categorized as class III, though this is somewhat controversial, with some authors classifying CIP as class II/IV. Unlike LEV or MOX, CIP presents a pH-dependent solubility. It is highly soluble at an acidic pH, however, at an intestinal pH of 6.8 to 7.5, its solubility is much lower [41]. Any meals or beverages able to significantly affect the pH may thus affect CIP oral bioavailability [42].

### 3.2. Distribution Process: Role of Patient´s Pathophysiological Characteristics

After entering systemic circulation, the drug must distribute throughout the body via the bloodstream to the tissues. The extent of drug distribution depends on a variety of factors, including the physicochemical properties of the drug, the rate of blood flow to the tissue, and the ability of the drug to bind to plasma proteins and tissue. Given that only unbound or free drugs can access the site of infection [43,44], the influence of plasma protein binding on the distribution of FQs was evaluated. The percent of plasma protein binding is low for FQs (30% for CIP [45], 31% for LEV [46], and 48% for MOX) [37]. Moreover, it has not been established that variability in plasma protein binding has any significant direct or indirect impact on the therapeutic effectiveness of FQs [47]. Regarding the tissue distribution, the physicochemical properties of FQs permit rapid penetration into extravascular and intracellular sites, with a rapid equilibrium established between compartments. CIP, LEV, and MOX are widely distributed throughout the body and reach high concentrations in a variety of tissues, such as the urinary tract (e.g., urine, prostate) [48,49], and other areas such as the lungs, paranasal sinuses, inflamed lesions, and bones [50,51].

**Figure 2:**
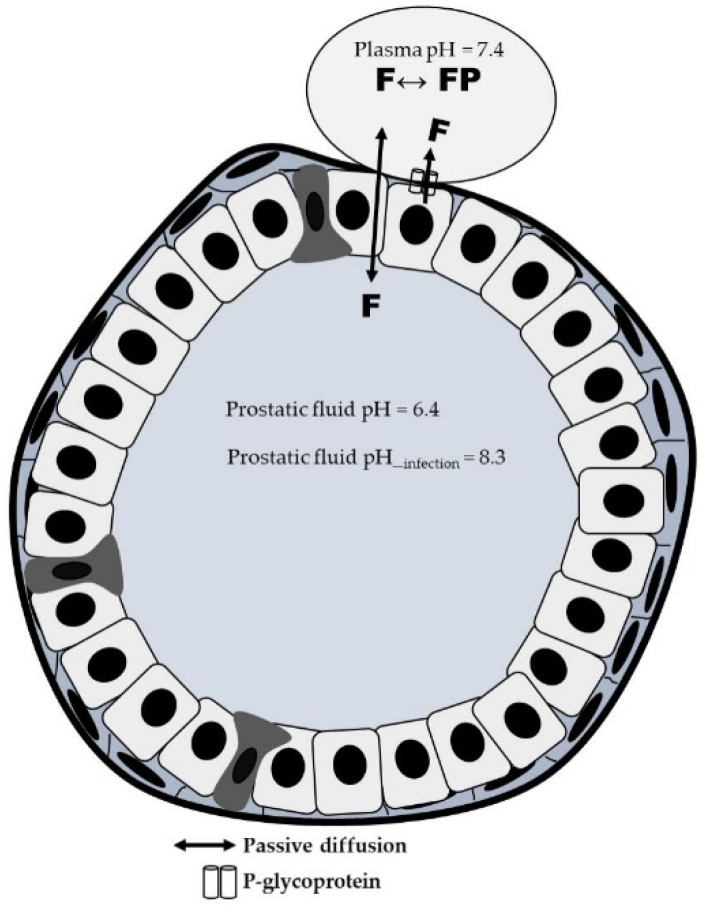
Distribution mechanisms of fluoroquinolone from the blood to the prostate gland. F: unbound fluoroquinolone; FP: protein-bound fluoroquinolone.

Specifically, FQs are effective in the treatment of many types of urological infections and other systemic infections due to their ability to achieve high concentrations in tissues and body fluids and their wide antibacterial spectrum. However, several complex mechanisms are involved in the penetration of special tissues, such as prostate. In addition to passive diffusion [52], conditioned by the drug’s acidic or alkaline nature, its pKa, and the pH of prostatic fluid, there is evidence of the involvement of efflux transporters–primarily P-glycoprotein–on FQs tissue penetration (Figure 2).

The results of Zimmermann et al. strongly support the role of efflux transporters on the prostatic tissue penetration of LEV [53], but not of CIP [54]. Due to this complexity, interindividual variability in drug penetration into tissues could result in variability of concentrations at the site of infection and condition the effectiveness of the treatment or affect to the emergence of bacterial resistance. Whole body physiologically based pharmacokinetic (PBPK) models provide a valuable tool to incorporate drug disposition characteristics–including the role of transporters–and predict unbound tissue distribution in different organs. The application of PBPK modeling has increased over the past decade to improve the mechanistic understanding of drug PK and support dosing recommendations [55]. PBPK models can also incorporate relevant disease-specific changes in the physiology, allowing the prediction of drug PK under different chronic conditions, as for example renal or hepatic disease, heart failure, or obesity [56,57,58].

Distribution studies use central blood/plasma concentrations as a surrogate for tissue distribution as they are easily accessible to measure. Volume of distribution (Vd) is the PK parameter that represents the degree to which a drug is able to distribute throughout the body to the tissues [59]. Body weight and its changes in obese patients, age, and pathological condition of patients can explain the interindividual variability of distribution seen with FQs to a certain degree. As can be observed in Table 1, when CIP was infused to a group of obese, the Vd was found to be 23% larger in the obese group than in the non-obese group. However, when the Vd was adjusted for the total body weight, the obese exhibited lower Vd/kg than the non-obese subjects. These findings indicate that CIP is not highly distributed into adipose tissue [60]. Additional population PK analysis studies conducted in elderly patients [61,62,63] and in adult patients with septic shock [64] revealed that total body weight is a significant covariate on the Vd of CIP. No significant changes in Vd have been found in patients with hepatic or renal impairment [65,66,67]. A high variability in this parameter was observed in patients with critical illness, but no covariates were associated with this variability due the complex of the pathology [62,64,68,69]. For LEV, patient-specific factors such as age, sex, and race [70], but not obesity–even considering obese patients and severely morbidly obese [71,72]–contributed to variability in Vd (Table 2). The Vd of MOX, however, was not significantly affected by age or sex [73], but was found to be correlated with lean body weight for both normal weight [74] and obese patients [75], as has been published in the articles referenced in Table 3.

### 3.3. Elimination Process: Role of Renal and Hepatic Function

FQs are eliminated from the body via two main mechanisms: biotransformation–or hepatic metabolism–and renal excretion. Once the antibiotic reaches the systemic circulation, these elimination processes function to decrease the blood concentration of FQs and consequently decrease antibiotic exposure at the site of infection [21,34]. Clearance (CL) is defined as the volume of body fluid, usually plasma, from which the drug is completely removed per unit of time. This PK parameter reflects the rate of drug elimination from the body and is proportional to the blood concentration of the drug. For every drug, each organ of elimination has its own clearance (e.g., hepatic clearance or renal clearance). The total body clearance of the drug is therefore the sum of the clearances from all eliminating organs (CL = CL_Renal_ + CL_Hepatic_ + CL_Other_) [59]. Clearance is the factor determining the average concentration of FQs after continuous intravenous infusion. After oral administration, however, the elimination process is determined by both the clearance and absorption process and underlying bioavailability (CL/F).

Different factors contribute to the interindividual variability in the CL of the urological FQs under review. First, Table 1 summarizes the changes in PK parameters related to CIP elimination processes (T_1/2_ and CL) and different patient subpopulations. For CIP, several mechanisms and factors may contribute to the interindividual variability in CL. Non-renal mechanisms of elimination–mainly hepatic metabolism–account for approximately one-third of CIP elimination. Four metabolites of CIP– desethyleneciprofloxacin, sulfo-ciprofloxacin, oxo-ciprofloxacin, and N-acetylciprofloxacin–have been recovered in the urine and feces. Due to changes in chemical structure, these metabolites have some antibacterial activity, but less than that of the parent compound [46]. Approximately 15% of a 100 mg intravenous dose of CIP is excreted in the feces, likely due to elimination directly through the intestinal mucosa and biliary excretion. The remaining two-thirds of the CIP dose is eliminated via the kidneys, due to a combination of glomerular filtration and tubular secretion [76]. As a result of undergoing CL through both non-renal and renal pathways, CIP has a relatively short half-life when compared to other FQs and requires twice daily dosing [39,46]. Population PK modeling has been used in several studies to estimate the effect of individual PK parameter values in a variety of patient populations and bacterial infections [61,62,64,68,69,77,78,79,80], showed in Table 1. Factors affecting renal and hepatic function could also be responsible for the interindividual variability in the CL of CIP, and their effect may be difficult to predict. Hepatic dysfunction appears to have minimal effect on the elimination of CIP, with no changes in CL found in chronic cirrhotic patients [65]. Creatinine clearance (CL_CR_), however, has been identified in multiple population PK studies as one of the main covariates responsible for interindividual variability in the systemic CL of CIP. In patients with varying degrees of renal dysfunction, CIP CL has been shown to decrease as CL_CR_ decreases [66,67,77]. Consequently, age-related decline in renal function could also lead to a reduction in CIP elimination in older adults [61,62,63]. In addition, an increase in CL has been reported in obese patients when compared to patients of normal weight, which could be related to the increase in glomerular filtration and tubular secretion known to occur in obese adults [60]. Lastly, critically ill patients exhibit higher interindividual variability in CL associated with pathophysiological changes driven by altered renal function [62,64,68,69,80]. Non-renal mechanisms, such as biliary clearance, may effectively compensate for the reduction in renal CL in these patients, and could further contribute to the increase in interindividual variability [64,80].

Approximately 83% of LEV is excreted in the urine as an unchanged drug, indicating that it primarily undergoes renal elimination [36,39]. Similarly to CIP, population PK modeling has been used in several studies with LEV to estimate the effect of individual PK parameter values in a variety of patient populations and bacterial infections [70,81,82,83,84,85,86,87,88,89,90,91,92,93] (Table 2). In several studies, CL_CR_ [71,82,83,84,85,91,92,93], age [70], and race [70] were found to be covariates that influenced the CL of LEV. In hospitalized elderly patients with varying degrees of renal function, CL_CR_ was again shown to be the main covariate associated with interindividual variability in LEV CL [90]. A prospective population PK study conducted in patients with bone and joint infections demonstrated that age and glomerular filtration rate were covariates related to interindividual variability of CL/F [81]. Critical illness was not a significant variable in altering LEV CL per se, with altered renal function being the determining factor [77,93,94,95,96]. Obesity may be another factor affecting the interindividual variability of LEV PK. However, most studies with LEV have been performed in normal weight patients, and only a few published studies performed in overweight and obese patients. One such study reported a higher CL of LEV in morbidly obese patients and suggested that CL was related to CL_CR_ estimated by the Cockcroft–Gault equation and ideal body weight [72].

MOX primarily undergoes hepatic metabolism and fecal excretion. Despite the large percentage of metabolism by the liver, moxifloxacin does not appear to be transformed by the cytochrome P450 (CYP) isoenzyme system, making it less susceptible to drug–drug interactions. Moxifloxacin has two metabolites, M1 (sulpho-compound) and M2 (glucuronide) [96,97,98]. Total clearance is modified only by lean body weight in healthy adults [37,73]. As shown Table 3, the PK after a single and multiple intravenous doses of MOX differed only marginally in patients with severe hepatic impairment compared to healthy patients, and demonstrated no accumulation [99]. Only 20% of MOX is excreted unchanged by the kidneys, conditioned by the processes of glomerular filtration and tubular reabsorption. As a result, renal impairment has little clinically relevant effect on the PK of MOX, including CL, and does not require dose adjustments [96]. MOX PK in critically ill patients with acute renal failure undergoing extended daily dialysis are similar to healthy patients without renal impairment [96,100,101]. Other patient-specific factors such as age [74], race [102], and obesity [75] have not been shown to be responsible for the interindividual variability in the CL of MOX.

FQs exhibit dose-independent PK, meaning that F, CL, and Vd are constant over the range of doses encountered clinically [39]. Several pathophysiological factors, that may be present in patients with urological infections, could influence interindividual variability in FQs PK and potentially affect clinical response and outcomes. Given their large Vd and ability to accumulate in tissues, interindividual variability in the Vd of FQs could affect the degree that FQs are able to penetrate the site of infection. Additional tissue distribution studies could therefore help to better understand variability in the Vd of FQs, especially associated with patients’ pathophysiological characteristics. However, the importance of CL is far more evident [103]. Given FQs’ concentration-dependent antibacterial activity, understanding the interindividual variability of CL after intravenous administration and F variability after oral administration is crucial to ensuring adequate antibiotic exposure–AUC–is achieved and maintained when treating urological infections. CL, especially for CIP and LEV, decreases fundamentally with decrease in renal function. This decrease in CL translates to a higher AUC in patients and, as a result, a higher probability of experiencing concentration-dependent adverse effects [104,105]. Another important aspect to consider is the impact of drug–drug interactions (DDIs) on drug exposure. PBPK modeling and simulation can be used as a tool to determine the impact of disease-related physiological changes and DDIs on the systemic exposure of FQs, and the possible need of dose adjustment in specific diseases and/or due to co-medications [106]. As an example, alterations in blood flow to main organs and decrease in clearance observed in chronic kidney disease or chronic heart failure can be incorporated in the model to predict changes in the ADME properties of FQs. In addition, mechanistic modeling can be used to explore possible disease effects, test hypotheses, and generate supporting evidences when not enough clinical data are available [107].

**Table 1:** Steady-state pharmacokinetic parameters for **ciprofloxacin** in patients with several physiopathology conditions after intravenous or oral administration (values expressed as mean (standard deviation)).

Ciprofloxacin				
Study Characteristic	Vd (L)	Cl (L/h)	T_½_ (h)	Reference
**Healthy, non-obese**				
200 mg Infusion IV 21–30 years	199.1 (34.2)	26.8 (5.7)	4.2 (0.8)	Plaisance et al., 1987 [35]
	219.0 (35.8)	44.6 (7.2)	4.0 (0.3)	Allard et al., 1993 [60]
	146.0 (27.4)	25.2 (5.8)	4. 4(0.9)	Drusano et al., 1986 [77]
750 mg Oral				
21–29 years	256.0 (80.0) ^1^	29.5 (5.9) ^1^	5.2 (0.7)	Plaisance et al., 1987 [35]
46–68 years	217.0 (45.0) ^1^	50.4 (14.4) ^1^	3.7 (0.4)	Drusano et al.,1986 [77]
**Healthy, obese**				
400 mg Infusion IV 29 ± 7 years BMI = 36 ± 4 kg/m^2^	269.1 (51.6)	53.8 (9.5)	4.3 (0.6)	Allard et al., 1993 [60]
**Patients with cirrhosis**				
750 mg Oral 52 ± 6 years	218.1 (45.4) ^1^	45.9 (14.1) ^1^	3.7 (0.4)	Frost et al., 1989 [65]
**Patients with renal disease**				
200 mg Infusion IV 22–62 years				
CL_CR_ ≥ 100 mL/min	191.7 (35.4)	26.8 (5.7)	4.3 (0.8)	Drusano et al., 1987 [66]
CL_CR_ = 86–60 mL/min	243.0 (97.1)	26.3 (10.3)	6.1 (1.6)	
CL_CR_ = 11–57 mL/min	183.2 (47.7)	15.0 (3.8)	7.7 (1.2)	
CL_CR_ = 0 mL/min	210.2 (70.8)	15.4 (4.3)	8.5 (3.3)	
750 mg Oral 48–90 years				
CL_CR_ ≥ 50 mL/min	158.0 (46.5) ^1^	70.4 (48.9) ^1^	3.5 (1.2)	Gasser et al., 1987 [67]
CL_CR_ < 50 mL/min	113.8 (34.2) ^1^	29.4 (6.4) ^1^	6.3 (3.2)	
**Elderly patients**				
200 mg Infusion IV 78 ± 11 years Cl_CR_ = 45 ± 16 mL/min	100.8 (37.8)	16.6 (6.8)	5.8 (2.4)	Hirata et al., 1989 [63]
200 mg Infusion IV 73 ± 11 years CL_CR_ = 45 ± 16 mL/min	(61.0–118.0)	18.4 (4.5)	ND	Cios et al., 2014 [61]
**Acutely ill patients**				
200–400 mg Infusion IV 24–91 years Cl_CR_ = 63 ± 30 mL/min	111.0 (33.0)	17.0 (6.6)	ND	Forrest et al., 1993 [25]
400–1200 mg Infusion IV 56–71 years GFR = 32–101 mL/min	255.0 (51.0)	25.4 (67.8)	ND	Abdulla et al., 2020 [68]
400 mg Infusion IV 23–79 years Cl_CR_ =13–204 mL/min	107.5 (21)	18.6 (18.7)	ND	Li et al., 2019 [80]
400–600 mg Infusion IV 24–89 years Cl_CR_ = 7–204 mL/min	ND	15.2 (42.9)	ND	Roberts et al., 2019 [64]
400 mg Infusion IV 55–77 years	160 (51.2)	10.7 (46.9)	ND	Roger et al., 2016 [79]
200–400 mg Infusion IV 30–87 years GFR = 23–208 mL/min	ND	20.3 (51.2)	ND	Gieling et al., 2020 [69]

Vd: volume of distribution in steady state; Cl: systemic clearance; BMI: body mass index calculated as: body weight [in kilograms]/height^2^ [in meters]; GFR: Glomerular filtration rate (mL/min) by MDRD (MDRD: Modification of Diet in Renal Disease Study Group developed a four-variable formula to estimate the GFR); ClCr: creatinine clearance; T_½_: elimination half-life; D: unpublished data. ^1^ value of apparent volume of distribution in steady state (Vd/F) and apparent clearance (Cl/F), respectively.

**Table 2:** Steady-state pharmacokinetic parameters for levofloxacin in patients with several physiopathology conditions after intravenous or oral administration (values expressed as mean (standard deviation) or mean (range) when standard deviation is not published).

Levofloxacin				
Study Characteristic	Vd (L)	Cl (L/h)	T_½_ (h)	Reference
**Healthy young volunteers**				
500 mg Oral 22–36 years Cl_CR_ = 90–117 mL/min	90.6 (11.9) ^1^	9.5 (1.7) ^1^	7.0 (0.8)	Chien et al., 1997 [36]
**Healthy elderly volunteers**				
500 mg Oral 66–75 years Cl_CR_ = 47–80 mL/min	70.8 (8.4) ^1^	7. 3 (1.9) ^1^	7.6 (2.0)	Chien et al., 1997 [36]
**Patients with respiratory, urinary, and other infections**				
250–500 mg Infusion IV 47 ± 18 years Cl_CR_ = 86 ± 31 mL/min	ND	9.3 (4.3)	ND	Preston et al., 1998 [70]
**Patients adults with pulmonary tuberculosis**				
1000 mg Oral 30–54 years Cl_CR_= 51–125 mL/min	(33.5–114.5) ^1^	7.6 (1.5–19.2) ^1^	ND	Peloquin et al., 2008 [82]
**Patients with bone and joint infections**				
750 mg Oral 57 ± 20 years BW = 72 ± 16 kg Cl_CR_= 120 ± 74 mL/min	90.6 ^1^ (0.06)	6.10 (0.17) ^1^	ND	Eloy et al., 2020 [81]
**Obese patients**				
750 mg Infusion IV 18–55 years BMI (kg/m^2^) = 49.3 ± 20.7 Cl_CR_ = 140 ± 64 mL/min	83.8 (21.6)	9.8 (4.2)	5.9 (3.5)	Cook et al., 2011 [71]
**Acutely hospitalized older patients with several degrees of renal function**				
125–750 mg Oral 81 ± 28 years Cl_CR_ = 18–50 mL/min	ND	2.53 (1.46) ^1^	ND	Cojutti et al., 2017 [90]
**Intensive Care Unit**				
Acute renal failure 500 mg Infusion IV 33–62 years	114.0 (74–155)	3.1 (2.9–3.2)	34.5 (21.2–47.7)	Czock et al., 2006 [96]
Acute renal failure 33–62 years	82.8 (50.0)	2.5 (0.9)	21.8 (5.5)	Hansen et al., 2001 [95]
Criticall ill in continuous hemodiafiltration 500 mg Infusion IV 59 ± 6 years Cl_CR_ = 70 ± 67 mL/min	ND	3.6 (0.4)	ND	Wada et al., 2015 [91]
Continuous veno-venous hemofiltration 250 mg Infusion IV 23–70 years	ND	1.8–3.6	ND	Malone et al., 2001 [93]
Continuous veno-venous hemofiltration 500 mg Infusion IV 68 ± 5 years	105.7 (36.4)	3.26 (1.4)	28.0 (4.5)	Guenter et al., 2002 [94]

Vd: volume of distribution in steady state; Cl: systemic clearance; BW: body weight (kg); BMI: body weight [in kilograms]/height^2^ [in meters]; ClCr = creatinine clearance; T_½_: elimination half-life. ND: unpublished data. ^1^ value of apparent volume of distribution in steady state (Vd/F) and apparent clearance (Cl/F), respectively.

**Table 3:** Steady-state pharmacokinetic parameters for moxifloxacin in patients with several pathophysiologic conditions after intravenous or oral drug administration. Values expressed as mean (standard deviation) or mean (range) when standard deviation is not published.

Moxifloxacin				
Study with	Vd (L)	Cl (L/h)	T_½_ (h)	Reference
**Healthy volunteers**				
200 mg Oral 33 ± 5 years	222.0 (1.2) ^1^	13.1 (0.1) ^1^	11.8 (1.2)	Stass et al., 1998 [73]
400 mg Oral 18–46 years	175.9 (19.4) ^1^	101.0 (2.1) ^1^	ND	Grosjean et al., 2012 [74]
**Morbidly obese** patients BMI > 40 kg/m^2^ 400 mg Infusion IV 41 ± 12 years	165.0 (30.0)	9.6 (2)	12.2 (2.2)	Keess et al., 2011 [75]
Hospitalized **severe liver insufficiency** with pneumonia or spontaneous bacterial peritonitis 400 mg Infusion IV 40–78 years	154.1 (118.5–216.1)	8.8 (6.4–10.5)	10.4 (8.5–16.0)	Barth et al., 2008 [99]
Outpatients with pneumonia receiving **hemodialysis** 400 mg Oral 47–78 years	ND	6.5 (1.9) ^1^	ND	Tokimatsu et al., 2017 [101]
**Critical ill** patients receiving continuous hemodiafiltration 400 mg IV infusion 25–76 years	266 (154–514)	15.7 (8.1–49.39)	12.3 (3.7–34.0)	Czock et al., 2006 [96]
Intensive care unit with COPD ^2^ 400 mg Infusion IV 70 ± 10 years	115.0 (40.0)	8.85 (2.6)	9.7 (3.7)	Sionidou et al., 2019 [100]

Vd: Volume of distribution in steady state; Cl: Systemic clearance; T_½_: elimination half-life; IV: Intravenous administration; BMI: Body mass index, defined as body weight [in kilograms]/height^2^ [in meters]; ND: Unpublished data. ^1^ Value of apparent volume of distribution in steady state (Vd/F) and apparent clearance (Cl/F), respectively. ^2^ COPD: Chronic Obstructive Pulmonary Disease.

## 4. Antibacterial Activity of FQs: Interregional Variability in Pharmacodynamic Properties

When FQs reach the site of infection at an appropriate concentration and remain there for sufficient time, they interact with the microorganism, resulting in an antibacterial effect (Figure 1) [108]. The antibacterial effect is related to the specific spectrum of activity of each FQ. CIP has the most potent activity against Gram (-) bacteria–including *Enterobacteriaceae* and *P. aeruginosa*–and atypical bacteria, with little to no activity against Gram (+) bacteria. LEV and MOX retain activity against Gram (-) and atypical bacteria similar to CIP, but expand coverage to include certain Gram (+) bacteria, such as *S. pneumoniae* [46]. In addition, MOX is active against anaerobic bacteria. The broad spectrum of activity makes FQs highly effective against a wide variety of acute and chronic bacterial infections, including urological infections [2,3,4,109].

MIC is the most relevant PD parameter to define the potential inhibitory activity of an antimicrobial against a microorganism. Antibiotic susceptibility rates for bacterial pathogens are available through the European Committee on Antimicrobial Susceptibility Testing (EUCAST) guidelines (European Society of Clinical Microbiology and Infectious Diseases) [110], the American Clinical and Laboratory Standards Institute (CLSI) guidelines [111], and local databases.

MIC distribution could vary according to whether the microorganisms are sensitive, intermediate, or resistant to the antibiotic [11,13]. Resistance to FQs can occur through various mutational mechanisms, including alterations in the target enzymes, DNA gyrase and topoisomerase IV, or in the permeability of the cytoplasmic membrane and expression of efflux pumps and proteins [46]. Global surveillance studies demonstrated that FQs resistance rates increased in the past years in almost all bacterial species [112]. This has led to the development of many antimicrobial surveillance programs, such as the SENTRY Antimicrobial Surveillance Program [113], the Center for Disease Control and Prevention’s National Healthcare Safety Network (CDC NHSN) [114], and the European Antimicrobial Resistance Surveillance Network (EARS-Net) [115], which are essential in the fight against emerging resistance. These programs provide information on microorganism frequency and distribution and antimicrobial resistance trends in different geographical regions and in nosocomial and community-acquired infections using information from medical centers worldwide by antimicrobial susceptibility testing in a central laboratory. This information has the potential to guide therapeutic approaches for serious infections and may have value in the prevention and control of infection [114]. Due to the ongoing emergence of antibiotic resistance, it is critical to take into account the local MIC values and susceptibility patterns for different microorganisms, and their time-dependent evolution, as they are responsible for PD variability of FQs [11,13]. An increase in the use of FQs for the treatment of infections caused by *P. aeruginosa* has led to reductions in susceptibility rates by agent and by geographical region, with consequences in microbiological and clinical outcomes. Rates of resistance of *P. aeruginosa* strains to CIP ranged from 23.2% in North America, 29.7% in Europe, 17.8% in Asian-Pacific, and 40.3% in Latin America between 1997 and 2016 [116]. *E. coli* is responsible for causing multiple urological infections, and the development of FQs-resistant strains could have a significant impact on clinical efficacy and outcomes in the treatment of these infections [117,118,119]. Resistance rates of *Neisseria gonorrhoeae* to FQs are highly variable, with rates in Asia as high as 40% to 100%, whereas resistance rates in Europe and North America range from <10% in rural areas to >30% in established sexual networks [112]. Higher rates of FQs resistance are expected in intensive care units (ICUs) due to the multiple factors, including frequent use of broad-spectrum antibiotics, multitude of invasive procedures, and increased likelihood of multidrug resistant pathogen transmission. For CIP and LEV, rates of resistance to *E. coli*, *Klebsiella* spp., *Enterobacter* spp., and *P. aeruginosa* were shown to be 35%, 12%, 9%, and 32%, respectively, in the United States, and 24%, 25%, 24%, and 39%, respectively, in Europe [119,120,121].

Differences in the MIC distribution for a specific bacterial pathogen could have a key role in the clinical and microbiological response after a standard empiric dose [14,16,17]. Since the selection of an antimicrobial therapy and its dose should be guided based on local susceptibility and resistance patterns, there is a critical need for determination of current antibacterial resistance rates and their time-dependent evolution at a local scale for urological infections [17,119]. However, in addition to the local susceptibility profile, the PK/PD analysis that allows to estimate the CFR should also be considered to optimize the antimicrobial dosing selections for clinical decision making. In fact, susceptibility data alone are not always useful for detecting changes in the likelihood of treatment success [122,123].

FQs susceptibility rates can vary widely for different bacterial pathogens, which can affect the ability to achieve the PK/PD indices necessary for clinical and microbiological cure after a drug dose, without ignoring the individual patient’s PK [114]. Several MCS analyses demonstrated that a 400 mg intravenous dose of CIP given every 12 h to critically ill patients achieved a PTA > 90% for an AUC/MIC ≥ 125 for isolates with an MIC of 0.25 mg/L. However, the PTA decreased to 50% and 10% as the MICs increased to 0.5 mg/L and 1 mg/L, respectively, for Gram (-) bacteria [25]. Another study demonstrated FQs treatment failure rates of 0.8% for *E. coli* isolates with an MIC of ≤0.12 mg/L compared to 6.9% for isolates with an MIC of >0.12 mg/L to ≤2 mg/L in adult female patients with complicated urinary tract infections [27].

**Figure 3:**
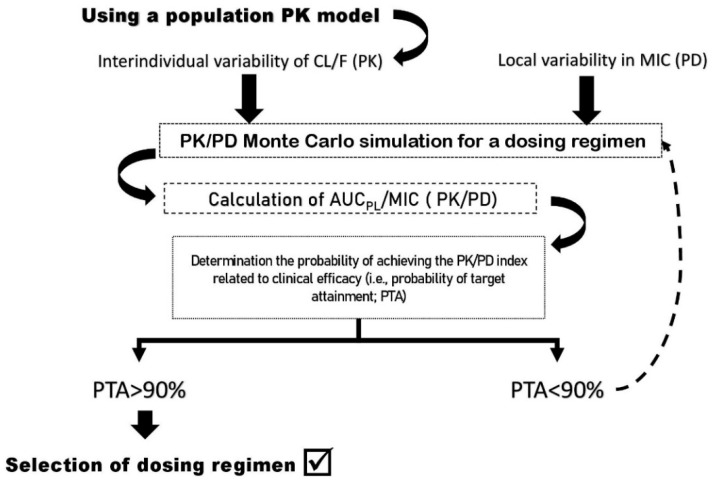
Model-based approach to select FQ dosing regimen based on % probability of target attainment. Scheme of the steps needed to apply PK/PD analysis and Monte Carlo simulation in clinical setting. PK: Pharmacokinetics; PD: Pharmacodynamics; CL: Clearance; F: Biodisponibility; AUC_PL_: area under the plasma drug concentration-time curve from time 0–24 h; MIC: minimum inhibitory concentration; PTA: Probability of target attainment.

Considering FQs’ interindividual pharmacological variability, developing urological patient-tailored effective dosing strategies in order to improve microbiological and clinical outcome may be necessary [13,19,124], as is proposed in Figure 3.

## 5. Conclusions

Due to a broad antimicrobial spectrum of activity and a favorable tissue penetration at the site of infection, fluoroquinolones (FQs) are a critical group of antimicrobials prescribed in urological infections, especially in prostatitis where they are life-saving [2]. However, urological FQs, including CIP, LEV, and MOX, present an important interindividual variability in PK associated with patient-specific characteristics. Thus, differences in interregional microorganism frequency, distribution, and resistance patterns could be encountered in clinical practice. This review highlights the need to take into account FQs’ interindividual pharmacological variability to develop urological patient-tailored effective dosing strategies in order to improve microbiological and clinical outcomes, prevent the emergence of resistance, and minimize the incidence of adverse effects.

## References

[1] World Health Organization (2018). Critically Important Antimicrobials for Human Medicine, 6th revision.

[2] Gray C., Loshak H. (2019). Fluoroquinolones for the treatment of intra-abdominal infections. CADTH Rapid Response Report: Summary with Critical Appraisal.

[3] Morales D.R., Slattery J., Pinheiro L., Kurz X., Hedenmalm K. (2018). Indications for Systemic Fluoroquinolone Therapy in Europe and Prevalence of Primary-Care Prescribing in France, Germany and the UK: Descriptive Population-Based Study. Clin. Drug Investig..

[4] Bonkat G., Bartoletti R., Bruyére F., Cai T., Geerlings S.E., Köves B., Schubert S., Pilatz A., Veeratterapillay R., Wagenlehner F. (2020). European Association of Urology: Guidelines on Urological Infections.

[5] Stamatiou K., Pierris N. (2017). Mounting resistance of uropathogens to antimicrobial agents: A retrospective study in patients with chronic bacterial prostatitis relapse. Investig. Clin. Urol..

[6] Zowawi H.M., Harris P.N., Roberts M.J., Tambyah P.A., Schembri M.A., Pezzani M.D., Williamson D.A., Paterson D.L. (2015). The emerging threat of multidrug-resistant Gram-negative bacteria in urology. Nat. Rev. Urol..

[7] European Medicines Agency Fluoroquinolone and Quinolone Antibiotics: PRAC Recommends Restrictions on Use New Restrictions Follow Review of Disabling and Potentially Long-Lasting Side Effects. https://www.ema.europa.eu/en/documents/press-release/fluoroquinolone-quinolone-antibiotics-prac-recommends-restrictions-use_en.pdf.

[8] FDA Drug Safety Communication: FDA Advises Restricting Fluoroquinolone Antibiotic Use for Certain Uncomplicated Infections 2016. https://www.fda.gov/drugs/drug-safety-and-availability/fda-drug-safety-communication-fda-advises-restricting-fluoroquinolone-antibiotic-use-certain.

[9] Bonkat G., Pilatz A., Wagenlehner F. (2019). Time to Adapt Our Practice? The European Commission Has Restricted the Use of Fluoroquinolones since March 2019. Eur. Urol..

[10] Almalki Z.S., Yue X., Xia Y., Wigle P.R., Guo J.J. (2017). Utilization, Spending, and Price Trends for Quinolones in the US Medicaid Programs: 25 Years’ Experience 1991–2015. PharmacoEconomics Open.

[11] Sumi C.D., Heffernan A.J., Lipman J., Roberts J.A., Sime F.B. (2019). What Antibiotic Exposures Are Required to Suppress the Emergence of Resistance for Gram-Negative Bacteria? A Systematic Review. Clin. Pharmacokinet..

[12] Martinez M.N., Papich M.G., Drusano G.L. (2012). Dosing regimen matters: The importance of early intervention and rapid attainment of the pharmacokinetic/pharmacodynamic target. Antimicrob. Agents Chemother..

[13] Heffernan A.J., Sime F.B., Lipman J., Roberts J.A. (2018). Individualising Therapy to Minimize Bacterial Multidrug Resistance. Drugs.

[14] Asin-Prieto E., Rodriguez-Gascon A., Isla A. (2015). Applications of the pharmacokinetic/pharmacodynamic (PK/PD) analysis of antimicrobial agents. J. Infect. Chemother..

[15] Ambrose P.G., Bhavnani S.M., Rubino C.M., Louie A., Gumbo T., Forrest A., Drusano G.L. (2007). Pharmacokinetics-pharmacodynamics of antimicrobial therapy: It’s not just for mice anymore. Clin. Infect. Dis..

[16] Onufrak N.J., Forrest A., Gonzalez D. (2016). Pharmacokinetic and Pharmacodynamic Principles of Anti-infective Dosing. Clin. Ther..

[17] Bonkat G., Wagenlehner F. (2019). In the Line of Fire: Should Urologists Stop Prescribing Fluoroquinolones as Default?. Eur. Urol..

[18] Mehvar R. (2006). Estimation of pharmacokinetic parameters based on the patient-adjusted population data. Am. J. Pharm. Educ..

[19] de Velde F., Mouton J.W., de Winter B.C.M., van Gelder T., Koch B.C.P. (2018). Clinical applications of population pharmacokinetic models of antibiotics: Challenges and perspectives. Pharmacol. Res..

[20] Mouton J.W., Dudley M.N., Cars O., Derendorf H., Drusano G.L. (2005). Standardization of pharmacokinetic/pharmacodynamic (PK/PD) terminology for anti-infective drugs: An update. J. Antimicrob. Chemother..

[21] Sy S.K., Zhuang L., Derendorf H. (2016). Pharmacokinetics and pharmacodynamics in antibiotic dose optimization. Expert Opin. Drug Metab. Toxicol..

[22] European Medicines Agency Guideline on the Use of Pharmacokinetics and Pharmacodynamics in the Development of Antimicrobial Medicinal Products. https://www.ema.europa.eu/en/documents/scientific-guideline/guideline-use-pharmacokinetics-pharmacodynamics-development-antimicrobial-medicinal-products_en.pdf.

[23] Bhavnani S.M., Forrest A., Hammel J.P., Drusano G.L., Rubino C.M., Ambrose P.G. (2008). Pharmacokinetics-pharmacodynamics of quinolones against *Streptococcus pneumoniae* in patients with community-acquired pneumonia. Diagn. Microbiol. Infect. Dis..

[24] Drusano G.L., Preston S.L., Fowler C., Corrado M., Weisinger B., Kahn J. (2004). Relationship between fluoroquinolone area under the curve: Minimum inhibitory concentration ratio and the probability of eradication of the infecting pathogen, in patients with nosocomial pneumonia. J. Infect. Dis..

[25] Forrest A., Nix D.E., Ballow C.H., Goss T.F., Birmingham M.C., Schentag J.J. (1993). Pharmacodynamics of intravenous ciprofloxacin in seriously ill patients. Antimicrob. Agents Chemother..

[26] Ambrose P.G. Quinolone In Vitro Susceptibility Test Interpretive Criteria Evaluations. The National Antimicrobial Susceptibility Testing Committee for the United States. USCAST 001, 15 October 2018. https://app.box.com/s/e14zs4u4tpxs02ppjb97czmckvbm99sg.

[27] Rattanaumpawan P., Nachamkin I., Bilker W.B., Roy J.A., Metlay J.P., Zaoutis T.E., Lautenbach E., CDC Prevention Epicenters Program (2017). High fluoroquinolone MIC is associated with fluoroquinolone treatment failure in urinary tract infections caused by fluoroquinolone susceptible *Escherichia coli*. Ann. Clin. Microbiol. Antimicrob..

[28] Peloquin C.A., Cumbo T.J., Nix D.E., Sands M.F., Schentag J.J. (1989). Evaluation of intravenous ciprofloxacin in patients with nosocomial lower respiratory tract infections. Impact of plasma concentrations, organism, minimum inhibitory concentration, and clinical condition on bacterial eradication. Arch. Intern. Med..

[29] Scaglione F., Esposito S., Leone S., Lucini V., Pannacci M., Ma L., Drusano G.L. (2009). Feedback dose alteration significantly affects probability of pathogen eradication in nosocomial pneumonia. Eur. Respir. J..

[30] Li Y., Le W.J., Li S., Cao Y.P., Su X.H. (2017). Meta-analysis of the efficacy of moxifloxacin in treating Mycoplasma genitalium infection. Int. J. STD AIDS.

[31] Noreddin A.M., Hoban D.J., Zhanel G.G. (2005). Comparison of gatifloxacin and levofloxacin administered at various dosing regimens to hospitalised patients with community-acquired pneumonia: Pharmacodynamic target attainment study using North American surveillance data for *Streptococcus pneumoniae*. Int. J. Antimicrob. Agents.

[32] Haeseker M., Stolk L., Nieman F., Hoebe C., Neef C., Bruggeman C., Verbon A. (2013). The ciprofloxacin target AUC: MIC ratio is not reached in hospitalized patients with the recommended dosing regimens. Br. J. Clin. Pharmacol..

[33] Trang M., Dudley M.N., Bhavnani S.M. (2017). Use of Monte Carlo simulation and considerations for PK-PD targets to support antibacterial dose selection. Curr. Opin. Pharmacol..

[34] Rizk M.L., Bhavnani S.M., Drusano G., Dane A., Eakin A.E., Guina T., Jang S.H., Tomayko J.F., Wang J., Zhuang L. (2019). Considerations for Dose Selection and Clinical Pharmacokinetics/Pharmacodynamics for the Development of Antibacterial Agents. Antimicrob. Agents Chemother..

[35] Plaisance K.I., Drusano G.L., Forrest A., Bustamante C.I., Standiford H.C. (1987). Effect of dose size on bioavailability of ciprofloxacin. Antimicrob. Agents Chemother..

[36] Chien S.C., Rogge M.C., Gisclon L.G., Curtin C., Wong F., Natarajan J., Williams R.R., Fowler C.L., Cheung W.K., Chow A.T. (1997). Pharmacokinetic profile of levofloxacin following once-daily 500-milligram oral or intravenous doses. Antimicrob. Agents Chemother..

[37] Stass H., Kubitza D. (1999). Pharmacokinetics and elimination of moxifloxacin after oral and intravenous administration in man. J. Antimicrob. Chemother..

[38] Nix D.E., Watson W.A., Lener M.E., Frost R.W., Krol G., Goldstein H., Lettieri J., Schentag J.J. (1989). Effects of aluminum and magnesium antacids and ranitidine on the absorption of ciprofloxacin. Clin. Pharmacol. Ther..

[39] Pitman S.K., Hoang U.T.P., Wi C.H., Alsheikh M., Hiner D.A., Percival K.M. (2019). Revisiting Oral Fluoroquinolone and Multivalent Cation Drug-Drug Interactions: Are They Still Relevant?. Antibiotics.

[40] Lee L.J., Hafkin B., Lee I.D., Hoh J., Dix R. (1997). Effects of food and sucralfate on a single oral dose of 500 milligrams of levofloxacin in healthy subjects. Antimicrob. Agents Chemother..

[41] Olivera M.E., Manzo R.H., Junginger H.E., Midha K.K., Shah V.P., Stavchansky S., Dressman J.B., Barends D.M. (2011). Biowaiver monographs for immediate release solid oral dosage forms: Ciprofloxacin hydrochloride. J. Pharm. Sci..

[42] Radwan A., Zaid A.N., Jaradat N., Odeh Y. (2017). Food effect: The combined effect of media pH and viscosity on the gastrointestinal absorption of ciprofloxacin tablet. Eur. J. Pharm. Sci..

[43] Calvo R., Lukas J.C., Rodriguez M., Leal N., Suarez E. (2006). The role of unbound drug in pharmacokinetics/pharmacodynamics and in therapy. Curr. Pharm. Des..

[44] Gonzalez D., Schmidt S., Derendorf H. (2013). Importance of relating efficacy measures to unbound drug concentrations for anti-infective agents. Clin. Microbiol. Rev..

[45] Aminimanizani A., Beringer P., Jelliffe R. (2001). Comparative pharmacokinetics and pharmacodynamics of the newer fluoroquinolone antibacterials. Clin. Pharmacokinet..

[46] Fish D.N., Chow A.T. (1997). The clinical pharmacokinetics of levofloxacin. Clin. Pharmacokinet..

[47] Bergogne-Bérézin E. (2002). Clinical role of protein binding of quinolones. Clin. Pharmacokinet..

[48] Drusano G.L., Preston S.L., Van Guilder M., North D., Gombert M., Oefelein M., Boccumini L., Weisinger B., Corrado M., Kahn J. (2000). A population pharmacokinetic analysis of the penetration of the prostate by levofloxacin. Antimicrob. Agents Chemother..

[49] Bulitta J.B., Kinzig M., Naber C.K., Wagenlehner F.M., Sauber C., Landersdorfer C.B., Sörgel F., Naber K.G. (2011). Population pharmacokinetics and penetration into prostatic, seminal, and vaginal fluid for ciprofloxacin, levofloxacin, and their combination. Chemotherapy.

[50] Kiang T.K., Hafeli U.O., Ensom M.H. (2014). A comprehensive review on the pharmacokinetics of antibiotics in interstitial fluid spaces in humans: Implications on dosing and clinical pharmacokinetic monitoring. Clin. Pharmacokinet..

[51] Gergs U., Ihlefeld D., Clauss T., Weiss M., Pönicke K., Hofmann G.O., Neumann J. (2018). Population Pharmacokinetics of Levofloxacin in Plasma and Bone of Patients Undergoing Hip or Knee Surgery. Clin. Pharmacol. Drug Dev..

[52] Wagenlehner F.M., Weidner W., Sörgel F., Naber K.G. (2005). The role of antibiotics in chronic bacterial prostatitis. Int. J. Antimicrob. Agents.

[53] Zimmermann E.S., Laureano J.V., Dos Santos C.N., Schmidt S., Lagishetty C.V., de Castro W.V., Dalla Costa T. (2015). Simultaneous Semimechanistic Population Analyses of Levofloxacin in Plasma, Lung, and Prostate To Describe the Influence of Efflux Transporters on Drug Distribution following Intravenous and Intratracheal Administration. Antimicrob. Agents Chemother..

[54] Zimmermann E.S., de Miranda Silva C., Neris C., Torres B.G.D.S., Schmidt S., Dalla Costa T. (2019). Population pharmacokinetic modeling to establish the role of P-glycoprotein on ciprofloxacin distribution to lung and prostate following intravenous and intratracheal administration to Wistar rats. Eur. J. Pharm. Sci..

[55] Sadiq M.W., Nielsen E.I., Khachman D., Conil J.M., Georges B., Houin G., Laffont C.M., Karlsson M.O., Friberg L.E. (2017). A whole-body physiologically based pharmacokinetic (WB-PBPK) model of ciprofloxacin: A step towards predicting bacterial killing at sites of infection. J. Pharmacokinet. Pharmacodyn..

[56] Rasool M.F., Ali S., Khalid S., Khalid R., Majeed A., Imran I., Saeed H., Usman M., Ali M., Alali A.S. (2021). Development and evaluation of physiologically based pharmacokinetic drug-disease models for predicting captopril pharmacokinetics in chronic diseases. Sci. Rep..

[57] Rasool M.F., Khalid S., Majeed A., Saeed H., Imran I., Mohany M., Al-Rejaie S.S., Alqahtani F. (2019). Development and Evaluation of Physiologically Based Pharmacokinetic Drug-Disease Models for Predicting Rifampicin Exposure in Tuberculosis and Cirrhosis Populations. Pharmaceutics.

[58] Gerhart J.G., Carreño F.O., Edginton A.N., Sinha J., Perrin E.M., Kumar K.R., Rikhi A., Hornik C.P., Harris V., Ganguly S. (2022). Best Pharmaceuticals for Children Act—Pediatric Trials Network Steering Committee Development and Evaluation of a Virtual Population of Children with Obesity for Physiologically Based Pharmacokinetic Modeling. Clin. Pharmacokinet..

[59] Derendorf H., Schmidt S. (2019). Rowland and Tozer’s Clinical Pharmacokinetics and Pharmacodynamics: Concepts and Applications.

[60] Allard S., Kinzig M., Boivin G., Sorgel F., LeBel M. (1993). Intravenous ciprofloxacin disposition in obesity. Clin. Pharmacol. Ther..

[61] Cios A., Wyska E., Szymura-Oleksiak J., Grodzicki T. (2014). Population pharmacokinetic analysis of ciprofloxacin in the elderly patients with lower respiratory tract infections. Exp. Gerontol..

[62] Forrest A., Ballow C.H., Nix D.E., Birmingham M.C., Schentag J.J. (1993). Development of a population pharmacokinetic model and optimal sampling strategies for intravenous ciprofloxacin. Antimicrob. Agents Chemother..

[63] Hirata C.A., Guay D.R., Awni W.M., Stein D.J., Peterson P.K. (1989). Steady-state pharmacokinetics of intravenous and oral ciprofloxacin in elderly patients. Antimicrob. Agents Chemother..

[64] Roberts J.A., Alobaid A.S., Wallis S.C., Perner A., Lipman J., Sjövall F. (2019). Defining optimal dosing of ciprofloxacin in patients with septic shock. J. Antimicrob. Chemother..

[65] Frost R.W., Lettieri J.T., Krol G., Shamblen E.C., Lasseter K.C. (1989). The effect of cirrhosis on the steady-state pharmacokinetics of oral ciprofloxacin. Clin. Pharmacol. Ther..

[66] Drusano G.L., Weir M., Forrest A., Plaisance K., Emm T., Standiford H.C. (1987). Pharmacokinetics of intravenously administered ciprofloxacin in patients with various degrees of renal function. Antimicrob. Agents Chemother..

[67] Gasser T.C., Ebert S.C., Graversen P.H., Madsen P.O. (1987). Ciprofloxacin pharmacokinetics in patients with normal and impaired renal function. Antimicrob. Agents Chemother..

[68] Abdulla A., Rogouti O., Hunfeld N.G.M., Endeman H., Dijkstra A., van Gelder T., Muller A.E., de Winter B.C.M., Koch B.C.P. (2020). Population pharmacokinetics and target attainment of ciprofloxacin in critically ill patients. Eur. J. Clin. Pharmacol..

[69] Gieling E.M., Wallenburg E., Frenzel T., de Lange D.W., Schouten J.A., Ten Oever J., Kolwijck E., Burger D.M., Pickkers P., Ter Heine R. (2020). Higher Dosage of Ciprofloxacin Necessary in Critically Ill Patients: A New Dosing Algorithm Based on Renal Function and Pathogen Susceptibility. Clin. Pharmacol. Ther..

[70] Preston S.L., Drusano G.L., Berman A.L., Fowler C.L., Chow A.T., Dornseif B., Reichl V., Natarajan J., Corrado M. (1998). Pharmacodynamics of levofloxacin: A new paradigm for early clinical trials. JAMA.

[71] Cook A.M., Martin C., Adams V.R., Morehead R.S. (2011). Pharmacokinetics of intravenous levofloxacin administered at 750 milligrams in obese adults. Antimicrob. Agents Chemother..

[72] Pai M.P., Cojutti P., Pea F. (2014). Levofloxacin dosing regimen in severely morbidly obese patients (BMI >/=40 kg/m(2)) should be guided by creatinine clearance estimates based on ideal body weight and optimized by therapeutic drug monitoring. Clin. Pharmacokinet..

[73] Stass H., Dalhoff A., Kubitza D., Schuhly U. (1998). Pharmacokinetics, safety, and tolerability of ascending single doses of moxifloxacin, a new 8-methoxy quinolone, administered to healthy subjects. Antimicrob. Agents Chemother..

[74] Grosjean P., Urien S. (2012). Reevaluation of moxifloxacin pharmacokinetics and their direct effect on the QT interval. J. Clin. Pharmacol..

[75] Kees M.G., Weber S., Kees F., Horbach T. (2011). Pharmacokinetics of moxifloxacin in plasma and tissue of morbidly obese patients. J. Antimicrob. Chemother..

[76] de Vroom S.L., van Hest R.M., van Daalen F.V., Kuil S.D., Mathôt R.A.A., Geerlings S.E., Jager N.G.L. (2020). Pharmacokinetic/pharmacodynamic target attainment of ciprofloxacin in adult patients on general wards with adequate and impaired renal function. Int. J. Antimicrob. Agents.

[77] Drusano G.L., Plaisance K.I., Forrest A., Standiford H.C. (1986). Dose ranging study and constant infusion evaluation of ciprofloxacin. Antimicrob. Agents Chemother..

[78] Roberts J.A., Cotta M.O., Cojutti P., Lugano M., Della Rocca G., Pea F. (2015). Does Critical Illness Change Levofloxacin Pharmacokinetics?. Antimicrob. Agents Chemother..

[79] Roger C., Wallis S.C., Louart B., Lefrant J.Y., Lipman J., Muller L., Roberts J.A. (2016). Comparison of equal doses of continuous venovenous haemofiltration and haemodiafiltration on ciprofloxacin population pharmacokinetics in critically ill patients. J. Antimicrob. Chemother..

[80] Li X., Zoller M., Fuhr U., Huseyn-Zada M., Maier B., Vogeser M., Zander J., Taubert M. (2019). Ciprofloxacin in critically ill subjects: Considering hepatic function, age and sex to choose the optimal dose. J. Antimicrob. Chemother..

[81] Eloy G., Lebeaux D., Launay M., Fernandez-Gerlinger M.P., Billaud E., Douez E., Mainardi J.L., Bouyer B., Jullien V. (2020). Influence of Renal Function and Age on the Pharmacokinetics of Levofloxacin in Patients with Bone and Joint Infections. Antibiotics.

[82] Peloquin C.A., Hadad D.J., Molino L.P., Palaci M., Boom W.H., Dietze R., Johnson J.L. (2008). Population pharmacokinetics of levofloxacin, gatifloxacin, and moxifloxacin in adults with pulmonary tuberculosis. Antimicrob. Agents Chemother..

[83] Zhang J., Xu J.F., Liu Y.B., Xiao Z.K., Huang J.A., Si B., Sun S.H., Xia Q.M., Wu X.J., Cao G.Y. (2009). Population pharmacokinetics of oral levofloxacin 500 mg once-daily dosage in community-acquired lower respiratory tract infections: Results of a prospective multicenter study in China. J. Infect. Chemother..

[84] Kiem S., Ryu S.M., Lee Y.M., Schentag J.J., Kim Y.W., Kim H.K., Jang H.J., Joo Y.D., Jin K., Shin J.G. (2016). Population pharmacokinetics of levofloxacin in Korean patients. J. Chemother..

[85] Chien S.C., Chow A.T., Natarajan J., Williams R.R., Wong F.A., Rogge M.C., Nayak R.K. (1997). Absence of age and gender effects on the pharmacokinetics of a single 500-milligram oral dose of levofloxacin in healthy subjects. Antimicrob. Agents Chemother..

[86] Kervezee L., Stevens J., Birkhoff W., Kamerling I.M., de Boer T., Dröge M., Meijer J.H., Burggraaf J. (2016). Identifying 24 h variation in the pharmacokinetics of levofloxacin: A population pharmacokinetic approach. Br. J. Clin. Pharmacol..

[87] Nomura K., Fujimoto Y., Morimoto Y., Kanbayashi Y., Matsumoto Y., Taniwaki M. (2008). Population pharmacokinetics of levofloxacin as prophylaxis for febrile neutropenia. Intern. Med..

[88] van den Elsen S.H.J., Sturkenboom M.G.G., Van’t Boveneind-Vrubleuskaya N., Skrahina A., van der Werf T.S., Heysell S.K., Mpagama S., Migliori G.B., Peloquin C.A., Touw D.J. (2018). Population Pharmacokinetic Model and Limited Sampling Strategies for Personalized Dosing of Levofloxacin in Tuberculosis Patients. Antimicrob. Agents Chemother..

[89] Cao G., Zhang J., Wu X., Yu J., Chen Y., Ye X., Zhu D., Zhang Y., Guo B., Shi Y. (2013). Pharmacokinetics and pharmacodynamics of levofloxacin injection in healthy Chinese volunteers and dosing regimen optimization. J. Clin. Pharm. Ther..

[90] Cojutti P.G., Ramos-Martin V., Schiavon I., Rossi P., Baraldo M., Hope W., Pea F. (2017). Population Pharmacokinetics and Pharmacodynamics of Levofloxacin in Acutely Hospitalized Older Patients with Various Degrees of Renal Function. Antimicrob. Agents Chemother..

[91] Wada T., Kobayashi M., Ono Y., Mizugaki A., Katabami K., Maekawa K., Miyamoto D., Yanagida Y., Hayakawa M., Sawamura A. (2015). Pharmacokinetics and the optimal regimen for levofloxacin in critically ill patients receiving continuous hemodiafiltration. J. Intensive Care..

[92] Canouï E., Kerneis S., Morand P., Enser M., Gauzit R., Eyrolle L., Leclerc P., Contejean A., Zheng Y., Anract P. (2022). Oral levofloxacin: Population pharmacokinetics model and pharmacodynamics study in bone and joint infections. J. Antimicrob. Chemother..

[93] Malone R.S., Fish D.N., Abraham E., Teitelbaum I. (2001). Pharmacokinetics of levofloxacin and ciprofloxacin during continuous renal replacement therapy in critically ill patients. Antimicrob. Agents Chemother..

[94] Guenter S.G., Iven H., Boos C., Bruch H.P., Muhl E. (2002). Pharmacokinetics of levofloxacin during continuous venovenous hemodiafiltration and continuous venovenous hemofiltration in critically ill patients. Pharmacotherapy.

[95] Hansen E., Bucher M., Jakob W., Lemberger P., Kees F. (2001). Pharmacokinetics of levofloxacin during continuous veno-venous hemofiltration. Intensive Care Med..

[96] Czock D., Husig-Linde C., Langhoff A., Schopke T., Hafer C., de Groot K., Swoboda S., Kuse E., Haller H., Fliser D. (2006). Pharmacokinetics of moxifloxacin and levofloxacin in intensive care unit patients who have acute renal failure and undergo extended daily dialysis. Clin. J. Am. Soc. Nephrol..

[97] Sullivan J.T., Woodruff M., Lettieri J., Agarwal V., Krol G.J., Leese P.T., Watson S., Heller A.H. (1999). Pharmacokinetics of a once-daily oral dose of moxifloxacin (Bay 12-8039), a new enantiomerically pure 8-methoxy quinolone. Antimicrob. Agents Chemother..

[98] Nightingale C.H. (2000). Moxifloxacin, a new antibiotic designed to treat community-acquired respiratory tract infections: A review of microbiologic and pharmacokinetic-pharmacodynamic characteristics. Pharmacotherapy.

[99] Barth J., Jager D., Mundkowski R., Drewelow B., Welte T., Burkhardt O. (2008). Single- and multiple-dose pharmacokinetics of intravenous moxifloxacin in patients with severe hepatic impairment. J. Antimicrob. Chemother..

[100] Sionidou M., Manika K., Pitsiou G., Kontou P., Chatzika K., Zarogoulidis P., Kioumis I. (2019). Moxifloxacin in Chronic Obstructive Pulmonary Disease: Pharmacokinetics and Penetration into Bronchial Secretions in Ward and Intensive Care Unit Patients. Antimicrob. Agents Chemother..

[101] Tokimatsu I., Shigemura K., Kotaki T., Yoshikawa H., Yamamichi F., Tomo T., Arakawa S., Fujisawa M., Kadota J.I. (2017). A Prospective Study of the Efficacy, Safety and Pharmacokinetics of Enteral Moxifloxacin in the Treatment of Hemodialysis Patients with Pneumonia. Intern. Med..

[102] Hasunuma T., Tohkin M., Kaniwa N., Jang I.J., Yimin C., Kaneko M., Saito Y., Takeuchi M., Watanabe H., Yamazoe Y. (2016). Absence of ethnic differences in the pharmacokinetics of moxifloxacin, simvastatin, and meloxicam among three East Asian populations and Caucasians. Br. J. Clin. Pharmacol..

[103] Bulik C.C., Bader J.C., Zhang L., Van Wart S.A., Rubino C.M., Bhavnani S.M., Sweeney K.L., Ambrose P.G. (2017). PK-PD Compass: Bringing infectious diseases pharmacometrics to the patient’s bedside. J. Pharmacokinet. Pharmacodyn..

[104] Täubel J., Prasad K., Rosano G., Ferber G., Wibberley H., Cole S.T., Van Langenhoven L., Fernandes S., Djumanov D., Sugiyama A. (2020). Effects of the Fluoroquinolones Moxifloxacin and Levofloxacin on the QT Subintervals: Sex Differences in Ventricular Repolarization. J. Clin. Pharmacol..

[105] Bidell M.R., Lodise T.P. (2016). Fluoroquinolone-Associated Tendinopathy: Does Levofloxacin Pose the Greatest Risk?. Pharmacotherapy.

[106] Cicali B., Lingineni K., Cristofoletti R., Wendl T., Hoechel J., Wiesinger H., Chaturvedula A., Vozmediano V., Schmidt S. (2021). Quantitative Assessment of Levonorgestrel Binding Partner Interplay and Drug-Drug Interactions Using Physiologically Based Pharmacokinetic Modeling. CPT Pharmacomet. Syst. Pharmacol..

[107] Kim C., Lo Re V., Rodriguez M., Lukas J.C., Leal N., Campo C., García-Bea A., Suarez E., Schmidt S., Vozmediano V. (2021). Application of a dual mechanistic approach to support bilastine dose selection for older adults. CPT Pharmacomet. Syst. Pharmacol..

[108] Li J., Roberts J., Working Group of Anti-infective Pharmacology of the International Society of Antimicrobial Chemotherapy (ISAC) (2021). Antibiotic pharmacokinetics/pharmacodynamics: Where are we heading?. Int. J. Antimicrob. Agents.

[109] Magri V., Boltri M., Cai T., Colombo R., Cuzzocrea S., De Visschere P., Giuberti R., Granatieri C.M., Latino M.A., Larganà G. (2019). Multidisciplinary approach to prostatitis. Arch. Ital. Urol. Androl..

[110] European Committee on Antimicrobial Susceptibility Testing Antimicrobial Wild Type Distribution of Microorganism. https://mic.eucast.org/Eucast2/.

[111] Clinical Laboratory Standards Institute. https://clsi.org/.

[112] Dalhoff A. (2012). Global fluoroquinolone resistance epidemiology and implictions for clinical use. Interdiscip. Perspect. Infect. Dis..

[113] Fuhrmeister A.S., Jones R.N. (2019). The Importance of Antimicrobial Resistance Monitoring Worldwide and the Origins of SENTRY Antimicrobial Surveillance Program. Open Forum. Infect. Dis..

[114] National Healthcare Safety Network (NHSN) CDC Centers for Disease Control and Prevention, National Center for Emerging and Zoonotic Infectious Diseases (NCEZID), Division of Healthcare Quality Promotion (DHQP). https://www.cdc.gov/nhsn/index.html.

[115] European Antimicrobial Resistance Surveillance Network (EARS-Net) European Centre for Disease Prevention and Control. https://www.ecdc.europa.eu/en/about-us/partnerships-and-networks/disease-and-laboratory-networks/ears-net.

[116] Shortridge D., Gales A.C., Streit J.M., Huband M.D., Tsakris A., Jones R.N. (2019). Geographic and Temporal Patterns of Antimicrobial Resistance in *Pseudomonas aeruginosa* Over 20 Years From the SENTRY Antimicrobial Surveillance Program, 1997–2016. Open Forum Infect. Dis..

[117] Stewardson A.J., Vervoort J., Adriaenssens N., Coenen S., Godycki-Cwirko M., Kowalczyk A., Huttner B.D., Lammens C., Malhotra-Kumar S., Goossens H. (2018). Study Group Effect of outpatient antibiotics for urinary tract infections on antimicrobial resistance among commensal Enterobacteriaceae: A multinational prospective cohort study. Clin. Microbiol. Infect..

[118] Critchley I.A., Cotroneo N., Pucci M.J., Mendes R. (2019). The burden of antimicrobial resistance among urinary tract isolates of *Escherichia coli* in the United States in 2017. PLoS ONE.

[119] Wagenlehner F. (2020). Urogenital infections. World J. Urol..

[120] Sader H.S., Farrell D.J., Flamm R.K., Jones R.N. (2014). Antimicrobial susceptibility of Gram-negative organisms isolated from patients hospitalized in intensive care units in United States and European hospitals (2009–2011). Diagn. Microbiol. Infect. Dis..

[121] de Kraker M.E., Jarlier V., Monen J.C., Heuer O.E., van de Sande N., Grundmann H. (2013). The changing epidemiology of bacteraemias in Europe: Trends from the European Antimicrobial Resistance Surveillance System. Clin. Microbiol. Infect..

[122] Valero A., Isla A., Rodríguez-Gascón A., Calvo B., Canut A., Solinís M.Á. (2019). Pharmacokinetic/pharmacodynamic analysis as a tool for surveillance of the activity of antimicrobials against *Pseudomonas aeruginosa* strains isolated in critically ill patients. Enferm. Infecc. Microbiol. Clin..

[123] Valero A., Rodríguez-Gascón A., Isla A., Barrasa H., Del Barrio-Tofiño E., Oliver A., Canut A., Solinís M.Á. (2021). *Pseudomonas aeruginosa* Susceptibility in Spain: Antimicrobial Activity and Resistance Suppression Evaluation by PK/PD Analysis. Pharmaceutics.

[124] Kandil H., Cramp E., Vaghela T. (2016). Trends in Antibiotic Resistance in Urologic Practice. Eur. Urol. Focus.

